# The biopsychosocial model and hypothyroidism

**DOI:** 10.1186/1746-1340-13-5

**Published:** 2005-04-12

**Authors:** Benjamin T Brown, Rod Bonello, Henry Pollard

**Affiliations:** 1 Department of Health and Chiropractic, Macquarie University, Sydney, Australia

## Abstract

This paper comments on the role and emergence of the biopsychosocial model in modern medical literature and health care settings. The evolution of the biopsychosocial model and its close association with modern pain theory is also examined. This paper seeks to discuss the place of this model with respect to the management of hypothyroidism. This discussion represents a forerunner to a randomised control trial that will seek to investigate the effect of a biopsychosocial-based treatment regime on hypothyroidism.

## Method

A search through Medline, Meditext, PubMed, OVID, Science direct, Austats, CINAHL, Expanded Academic ASAP was performed using the key words: Biopsychosocial model, hypothyroidism, treatment, levothyroxine, thyroid.

## What is the Biopsychosocial model?

The biopsychosocial model depicts a health care concept that has evolved in close association with current pain theory. It has sought coexistence with the dominant biomedical model of health care, which describes 'disease' as a failure of or within the *soma*, resulting from infection, injury or inheritance [1]. The biomedical model has its roots in the Cartesian division between mind and body [2].

In 1977, Engel described a crisis that modern medicine and psychiatry were facing. Disease, from a biomedical perspective was described in somatic parameters alone, there was little or no room for psychological, social and behavioural dimensions of illness within this model. This made adherence to this framework very difficult. There were somatic and mental disorders that simply did not fit the biomedical model, and hence it was no longer sufficient for the scientific and social responsibilities of either medicine or psychiatry [2,3]. Engel set out to develop a new framework that would account for the biological, psychological and social dimensions of illness and disease. It was essential that this new model provide a basis for the understanding and treatment of disease, whilst taking into account the patient, his/her social context and the impact of illness on that individual from a societal perspective [4,5]. This represented the development of the biopsychosocial model [2].

The biopsychosocial model states that ill health and disease are the result of an interaction between biological, psychological and social factors. The biopsychosocial model makes the distinction between pathophysiological processes that cause disease and the client's perception of their health and the effects on it, called the illness [6]. It seeks to build upon the biomedical model. Biological indices are still held in high regard, however, they represent only one of the defining factors for the diagnosis and management of disease under a biopsychosocial framework [2]. The biopsychosocial model describes psychological and social effects of disease risk, prevention, treatment compliance, morbidity, quality of life and survival [4].

Situations paradoxically arise in medicine in which a person who feels well is described biochemically, as having 'disease'. In contrast, a client's laboratory findings may reveal no 'disease', however the client still feels unwell. The biopsychosocial model provides a conceptual framework for dealing with such situations [2].

Of late, a great deal of attention has been given to the factors involved in chronic pain and depression. It is research like this that has highlighted the need for a paradigm such as the biopsychosocial model in the management of conditions other than chronic pain. Following the success of various psychological and cognitive interventions in the reduction of somatic signs and symptoms associated with certain conditions such as irritable bowel syndrome (IBS), non-cardiac chest pain, fibromyalgia and rheumatoid arthritis (RA), research has set out to explore the role of psychosocial factors in the disease process [3,7,8].

The biopsychosocial model avoids a strong 'disease' focus and seeks to address the client and his or her illness [9]. Clients are helped not only with biological disruptions, but also with their capacity to deal with being ill. It is proposed that this approach may be of benefit in reducing the frequency of clinic visits, hospitalisations, laboratory investigations and use of pharmacological agents. Changes in ones ability to cope, inherent belief systems as well as behavioural and social processes associated with being ill may also be improved through the implementation of this model [10].

## The Evolution of the Biopsychosocial Model

The biopsychosocial model represents the evolution of the biomedical model, and aligns favourably with recent ideas in pain theory and pathophysiology [3].

Early theories concerning pain were consistent with the specificity theory, which described pain as the result of noxious stimuli or somatic pathology alone [11]. There was little or no place for the influence of psychological or social factors within these original theories [3].

The mid nineteen sixties saw the emergence of the gate control theory of pain [11,12]. The theory proposed by Melzack and Wall, represented a more concise model that took into account the multidimensional nature of pain, allowing for physiological factors and the role of the brain in the processing of nociceptive stimuli. The gate control theory depicts three dimensions of pain, a sensory-physiologic, motivational-affective and a cognitive evaluative dimension. Scope for the influence of environmental factors on pain also exists within the body of this theory. The gate control model also provides a basis for understanding the depression and cognitive and motivational shifts witnessed in chronic pain situations [3,11].

Melzack through his study of phantom limb pain (pain that is localised in the region of a deafferented body part, subsequent to the loss of a limb)[13] developed the neuromatrix model [3,14] of pain. This model builds upon the gate control model and represents one of the more recent ideas in pain theory. The neuromatrix theory proposes that the various dimensions of pain experience are the result of a neural network program called the neuromatrix. This neuromatrix is influenced by genetic factors in conjunction with cognitive, sensory and affective experiences, which are individual specific. This model states that the unified pain experience depicts an aggregate of information from somatosensory, limbic and thalamocortical pathways.

The end product is the cyclic processing of neural information into a characteristic pattern, known as the neurosignature. Melzack postulates that a neurosignature exists for all types of pain, mood and psychological states [3,14]. The neuromatrix is also described as having both static and dynamic qualities, meaning that the neurosignature can be influenced/modified through learning and experience [14-17].

Put into context, this model suggests that neurosignatures for pain and depression exist throughout the neuromatrix. These signatures cannot be erased, but can be altered if changes are made to the entire network [3,11,14].

The biopsychosocial model has emerged over the past two decades and has sought to expand upon disease paradigms and complement pain models. It states that in order to understand and manage ill health, pain and disease, one must take into account the influence of biological, psychological and social factors [7].

## Biological Factors

It is acknowledged that there are many conditions (eg osteoarthritis and rheumatoid arthritis)[3] in which the symptoms experienced by clients are strongly linked to peripheral factors such as, inflammation or cartilage damage. These represent examples of biological disruption under a biopsychosocial framework. Previously, the influence of psychological and social factors on these conditions was considered of little importance and treatment was directed to areas of somatic pathology or nociceptive input [3]. Historically, the underlying disease process could account for many if not all of the biological features of disease and ill health. In this instance a biomedical treatment strategy could be implemented with high efficacy. It is important to note that the biomedical model has not been replaced. There are a number of diseases that can be diagnosed and managed without any consideration of psychosocial factors (eg. legionnaires disease and toxic shock syndrome) [18].

In contrast, the existence of pain and discomfort in conditions such as fibromyalgia, irritable bowel syndrome (IBS) and noncardiac chest pain is strongly linked to central nervous system (CNS) disturbance (eg. altered central processing) and psychosocial factors [3,7,8]. From a biopsychosocial perspective, biological factors represent one determinant of ill health and disease. Biological disruption may exhibit inconsistent weighting amongst various conditions, but it represents an essential component of diagnosis and management under the biopsychosocial model [2].

## Psychological Factors

Research into chronic pain and depression has revealed a number of significant psychological factors [19]. Clients experiencing pain lasting for prolonged periods of time often display a series of maladaptive coping responses that can influence the pain experience [3,10]. These responses include catastrophizing, perceived low self-efficacy and perceived helplessness. Clients presenting with the above responses, often report higher pain levels than those subjects that do not display these responses [3,19,20].

Psychological intervention (eg pain-coping strategies, cognitive behavioural therapy {CBT}) aimed at these maladaptive patterns and behaviours has demonstrated high rates of success in reducing symptomatology and disease progression in certain chronic pain conditions [3,10,20].

One area pain researchers are currently exploring is the differences in information processing and recall bias between chronic pain sufferers who present with comorbid depression and those non-depressed pain groups. It appears that clients with chronic pain demonstrate a propensity for recalling the negative information regarding their condition, in the presence of co-morbid depression. Clients without depression demonstrate a recall bias for positive illness related information. These studies suggest that depressed patients with chronic pain may be processing information differently to the non-depressed groups [3].

Pincus and Morley propose a model to account for the bias in information processing (specifically cognitive bias) demonstrated in chronic pain sufferers [21]. It is postulated that bias is the result of intersection of three schemas representing pain, illness and self. Within these schemas is a store of information that can interact and influence the processing of information. The *pain *schema encompass sensory intensity, along with spatial and temporal features of pain. The schema accounts for the immediate properties of the pain experience. They depict the interruption of ongoing behaviour and the commencement of pain avoidance and recuperative behaviours.

The *illness *schema depict information relating to affective and behavioural consequences of illness (eg. goal attainment, both long and short term and quality of life etc.). The identity, timeframe, perceived causes and consequences along with control of a particular illness, make up the schema.

The schema representing *self *is described in a number of ways. The self can be explained as 'an organized cognitive structure within long-term memory, which may incorporate both general trait like information about the self, as well as specific behaviours' [21]. The self can be viewed as temporally dynamic processing and assimilating information throughout life [21].

It is stated that contemporary individuals are striving to meet their positive goals and avoid unwanted outcomes [21]. This is determined by components of the self-schema and their projections as to what they might become. Illness, pain and other significant life events have the potential to disrupt aspects of the self. For example repeated pain whilst performing a specific task can interfere with or result in failure to complete that task.

Variations in the state of a person may be explained by the interaction of these schema. Activation of elements from one particular schema has the potential to simultaneously activate components of another schema, in a process known as *enmeshment*. Consequently, the activation of one schema via a relatively innocuous stimulus following enmeshment, can elicit unwanted effects (eg illness behaviour associated with pain syndromes) [21].

Recent studies performed by Buer and Linton describe the importance of cognitions (fear-avoidance beliefs and catastrophizing) in chronic pain situations [22]. The model used by Buer and Linton describe fear-avoidance specifically, as a fear of movement /(re) injury, which predispose to catastrophizing and avoidance, which in turn could lead to disuse, disability and depressive symptoms [22].

## Social Factors

There exists a considerable amount of data on the social determinants of ill health and disease. One of the contributors to this body of knowledge has been the Whitehall studies. The Whitehall I study initiated in the late 1960's, examined mortality rates over 10 years among male British Civil Servants aged 20–64 [23]. The Whitehall study sought to expand on the issue of the social class grouping, and counter some of the problems associated with this topic. Participants were physically examined and asked to complete questionnaires regarding their jobs, smoking habits in conjunction with personal and family medical histories. Certain participants were asked about car ownership, physical activity at work and general leisure activity, which completed the list of socio-economic outcome measures. The results of the study depicted an inverse association between grade (level) of employment and mortality from CHD and a range of other causes was observed [23].

The Whitehall II study was set up to investigate the effect of social gradient on morbidity and mortality, including determinants such as, work characteristics and social support [24]. Measures taken into consideration were, grade of employment, depressive symptoms, physical functioning, psychosocial work characteristics, life events and material problems and health related behaviour. The results revealed that some risk factors contribute jointly to the inequalities witnessed in mental and physical health. The incidence of secondary psychological stress associated with physical ill health is more prevalent in lower employment grades. Work was deemed most important in inequalities in depressive symptoms amongst men. Amongst female respondents, work and material disadvantage were equally salient in explaining inequalities in depressive symptoms [24].

There are various social factors that have gained recognition in recent years with respect to influences on depression among pain patients. Patients who report high family conflict and low family cohesion often display higher depression levels. Likewise, patients with low socio-economic status and lower levels of education also exhibit higher depression scores. Research into this area highlights a number of studies in which psychological intervention involving spouses or caregivers resulted in reduced pain and psychological distress [3].

In a recent review, Truchon describes a set of socio-demographic factors that have featured in chronic disability (related to low back pain) studies over the last decade [25]. These include; age, sex, education, ethnic background and financial compensation. Ringel *et al *describes a diagnostic protocol for irritable bowel syndrome (IBS), encompassing social factors such as, break up of a close relationship, early life experience, familial dysfunctions and family environment. It is postulated that these factors may influence the development of symptoms, clinical expression, course of the disorder and the utilization of health services [7].

## Hypothyroidism

Hypothyroidism refers to any metabolic state that results from a decrease in the amount of circulating thyroid hormones in the body. Hypothyroidism can be classified based on its time of onset (congenital or acquired), severity (overt {clinical} or mild {subclinical}), and the degree of endocrine aberration (primary or secondary) [26]. Primary hypothyroidism follows a dysfunction of the thyroid gland itself, whereas secondary hypothyroidism results from the dysfunction of metabolic or messenger pathways associated with thyroid hormone production and metabolism [27-29]. Primary hypothyroidism is characterised by reduced free thyroxine (FT4) levels and elevated thyroid stimulating hormone (TSH) levels. Diagnosis of secondary hypothyroidism presents as a clinical challenge as TSH levels can be reduced, normal or slightly elevated. Evaluation of other pituitary hormones becomes necessary in this situation [27].

According to the 1995 report from the Australian Bureau of Statistics (ABS) 5.3/1000 males and 27.3/1000 females experience thyroid dysfunction in Australia [30]. This figure is higher in the elderly, postmenopausal women and various groups presenting with psychological dysfunction [31-33]. In the United States hypothyroidism is the second most common endocrine disorder and it has been estimated that 18/1000 members of the general population display decreased thyroid hormone levels [33]. Hypothyroidism and subclinical hypothyroidism are considered more common than their counterpart, hyperthyroidism [33,34]. Hypothyroidism is the most common pathological hormone deficiency [26].

## Aetiology

Hypothyroidism is caused by a variety of different states and conditions. Autoimmune and iatrogenic causes constitute the most common sources of reduced thyroid hormone levels [26] (see table 1).

**Table 1:** Aetiology of Hypothyroidism

**Category**	**Cause**
Autoimmune	*Hashimoto's Thyroiditis, Reidels Disease, previous Graves disease, de Quervains thyroiditis, postpartum thyroiditis, Downs syndrome, family history of autoimmune disease or associated disorders (vitiligo, adrenal insufficiency, diabetes mellitus type-1, Sjogren's, coeliac disease), Turner's syndrome, Multiple Sclerosis, primary pulmonary hypertension*
Nutritional	*Iodine deficiency, excess intake of goitrogens, excessive iodine intake*
Environmental	*Radiation Exposure, exposure to polybrominated and polychlorinated biphenyls and resorcinol*
Iatrogenic	*Radioactive iodine therapy, medical radiation exposure, total or subtotal thyroidectomy, drugs impairing thyroid function (amiodarone, thalidomide, betaroxine, lithium carbonate, stavudine, aminoglutethimide)*
Hypothalamic	*Hypothalamic or suprasellar mass, history of hypothalamic radiotherapy or surgery, disorders causing hypothalamic dysfunction (eg sarcoidosis, heamochromatosis)*
Pituitary	*Known pituitary tumour, other elements of hypopituitarism, manifestations of sellar mass, history of pituitary surgery or radiotherapy, history of head trauma*
Other	*Postpartum status*

## Signs and Symptoms of Hypothyroidism

Hypothyroidism manifests in a variety of different forms [26]. Young infants and children born with deficiencies in thyroid hormones are at risk of brain damage and mental retardation [35,36]. Technological advancement in hormone assays has revealed that hypothyroidism is a relative, rather than an absolute state [37]. In fact, a spectrum of thyroid hormone deficiency exists. This spectrum, in conjunction with individual differences, allows for a multitude of differing hypothyroid presentations [26].

Overt hypothyroidism refers to patients with elevated thyroid stimulating hormone (TSH) levels and low free thyroxine (T4) levels. The term 'subclinical hypothyroidism' has featured repeatedly in the literature in recent years. Subclinical hypothyroidism is characterised by elevated TSH concentrations associated with normal thyroxine (T4) and triiodothyronine (T3) serum levels. Subclinical hypothyroidism is further categorized with respect to the following guidelines: [29,38] (see table 2).

**Table 2:** Subclinical Hypothyroidism Grading System

**Grade**	**TSH levels**	**Thyroxine (T4) levels**
1	*TSH above normal limits of reference range*	*Normal*
2	*TSH; 10.1–20 mU/L*	*Normal*
3	*TSH > 20 mU/L*	*Normal*

A relatively exhaustive index of signs and symptoms is listed below. However, it is important to keep in mind that the manifestation of hypothyroidism is often far from the textbook presentation. The symptoms of hypothyroidism can be subtle and are often confused with the signs of aging [39,40] (see table 3).

**Table 3:** Signs and Symptoms of Hypothyroidism

**System**	**Symptoms**
Central Nervous System	*Depression, fatigue, lethargy, forgetfulness, decreased concentration, memory deficit, slow thinking, cold intolerance, nerve entrapment syndromes, decreased sweating, ataxia*
Musculoskeletal	*Muscular weakness, cramps, myalgia, arthralgia, and delayed relaxation phase of reflexes*
Cardiovascular	*Bradycardia, diastolic hypertension, increased serum cholesterol, raised triglycerides, raised low-density lipoproteins, ascites, hyperhomocysteinaemia*
Gastrointestinal	*Constipation*
EENT	*Puffy eyes, enlarged tongue, hearing impairment, goitre, hoarseness of voice, dysphagia, sore throat*
Genito-Urinary	*Infertility, menstrual irregularities, heavy bleeding, impotence, galactorrhea, hyperprolactinemia*
General	*Weight gain, dry and coarse skin, brittle hair and nails*
Radiological	*Pericardial and pleural effusions, pituitary gland enlargement*

## Treatment

The current treatment of choice for individuals suffering from hypothyroidism is supplementation using the synthetic thyroid hormone, levothyroxine. Levothyroxine is an artificial version of the naturally occurring thyroid hormone, thyroxine (T4). Patients are required to take 50–150 μg of levothyroxine daily for the rest of their lives [31,34]. Thyroid hormones are monitored every 6–12 months to ensure that hormone levels are being maintained within physiological norms [31,32,34,41].

## Hypothyroidism, Mood Disorders and Stress

Depression is one of the major symptoms associated with hypothyroidism. According to the DSM-IV, a person presenting with depression (major depressive episode) must either have a depressed mood or interest for two or more weeks [42]. This mood must represent a change from the person's normal mood; social, occupational, educational, or other important functioning must be negatively impaired by the change in mood. To make the diagnosis of major depressive episode a patient must exhibit a depressed mood or interest and four or more of the following symptoms; sleep increase/decrease, diminished interest in formerly compelling or pleasurable activities, guilt, low self esteem, poor energy, poor concentration, appetite increase/decrease, psychomotor agitation/retardation and suicidal ideation [42].

While depression is strongly associated with hypothyroidism, the exact mechanisms are not yet known [43-45]. Haggerty states that almost 100% of patients presenting with severe hypothyroidism, are found to have serious concurrent depression [37]. It is well established that patients presenting with a decreased thyroid status exhibit higher lifetime frequency of depression than euthyroid subjects [46]. Furthermore, patients with major depression have a poorer response to antidepressant medication if they are hypothyroid [47,48]. Subclinical hypothyroidism may also reduce the threshold for the occurrence of major depression [46,47].

Research has sought to explain the above findings via the concept of central and peripheral hypothyroidism [46]. It is postulated that central abnormalities in thyroid hormone economy will not necessarily manifest in static peripheral hormone assays [49]. Therefore, serum thyroid hormone levels may appear normal in the presence of a central deficiency.

Gunnarsson *et al *suggests that biological correlates may exist for some of the depressive symptoms of hypothyroidism [50]. These include CSF CCK-4 (an anxiogenic peptide) and trytophan (the precursor to serotonin), as well as serum thyroid hormone levels [50].

It has been suggested that central serotonergic activity is reduced in hypothyroid patients. It is also postulated that comparatively higher thyroid stimulating hormone (TSH) levels may be a predictor of lower serotonin mediated endocrine responses and the presence of clinical depression [44]. Depression is associated with a deficiency in brain serotonergic (5-HT) activity. Duval postulates that the changes witnessed in hypothalamic pituitary thyroid (HPT) axis hormones during major depressive episodes, may be regarded as compensatory changes in order to correct reduced central serotonergic activity [44].

Sullivan states that transthyretin, a thyroid carrier protein, exists in differing forms both centrally and peripherally [46]. This carrier protein appears reduced in the cerebrospinal fluid (CSF) of depressed patients, which prevents the transport of thyroid hormones to the brain [45,46,49]. Alterations in central nervous system (CNS) thyroid hormone levels have a major effect on the serotonergic, adrenergic and GABAergic systems. Sher states that the brain utilizes thyroid hormones differently to other organs, it appears especially sensitive to subtle thyroid insufficiency. This means that thyroid function may significantly affect mood, behaviour, and cognitive function [45].

Research into stress, immunity and the HPT axis has demonstrated some interesting findings. In a recent study investigating the effects of stress on rat brains, Friedman *et al *suggested that acute stress may alter the levels of thyroid hormone T3, but not T4 in the rat brain [51]. Bauer *et al *researched the effects of chronic stress on hormone secretion in human subjects [52]. Thyroid hormone concentrations were assessed in a group of 84 East German refugees suffering from various psychiatric disorders. The results demonstrated reductions in both TSH and thyroid hormone concentrations. It was postulated that these results reflect severe chronic stress as opposed to the effects of psychiatric illness or thyroid dysfunction [52]. Cremaschi *et al *analysed the effects of chronic stress on the thyroid axis and its influence on the immune response in animals. The results showed that the thyroid hormones could be influenced by chronic stress, in particular T3 concentrations. Furthermore it was postulated that changes in thyroid axis function may play a role in regulation of the immune response [53].

Bauer *et al *describe a study in which a significant number of patients with prophylaxis resistant affective disorders (bipolar depression, unipolar depression, schizoaffective disorder) improved after being given supra-physiological doses of synthetic thyroid hormone [54]. It was suggested that this method of intervention may represent a useful and well-tolerated maintenance treatment for patients presenting with these subcategories of mood disorders [54].

There is a multitude of evidence highlighting the importance of the HPT axis in depression [37,49]. Alterations in thyroid hormones witnessed in mood disorders are unquestionable and this increased understanding of the role of thyroid hormones has lead to improvements in the management of depression [48]. There is however, evidence to dispute the relationship between thyroid dysfunction and mood disorders. The state of the literature as a whole suggests an association between thyroid dysfunction and depressive disorder, however the mechanism is unclear [55]. Engum *et al *describe a study of a large randomly selected population group examining the risk of anxiety disorders or depression in individuals with thyroid dysfunction. The results of this study showed a higher prevalence of depression in groups with previously known thyroid disorders, with lower prevalence in those with more recently diagnosed thyroid dysfunction. This study demonstrated a weak association between thyroid disorder and symptoms of anxiety and depression. Based on the results of their study Engum *et al *suggests that when depression and anxiety disorders are diagnosed in thyroid dysfunction sufferers, they should be treated as separate entities, and considered that these common conditions can occur and coexist without influencing each other [55]. Baldini *et al *described similar findings in a study of psychopathology and subclinical hypothyroidism. It was stated that when interfering factors related to individual vulnerability to depression and perception of disease were excluded, there was no direct correlation between subclinical hypothyroidism and mood disorders [56].

Fountoulakis *et al *suggests that the information available on hypothyroidism and depression does not demonstrate a causal relationship [57]. Stating that overt thyroid dysfunction is uncommon in depressed patients. Instead those authors suggest the presence of an underlying autoimmune process, which may affect thyroid function in depressed populations [57].

## The Biopsychosocial Model and Hypothyroidism

The biopsychosocial model represents a health concept. It depicts a treatment paradigm that acknowledges the contribution of biological, psychological and social factors in the disease process [2]. Alonso states that the biopsychosocial model is gaining acceptance within academic and institutional contexts [1]. This change however is not necessarily being reflected in the practical areas of medicine. The biopsychosocial model has been used to obtain a better understanding of the disease process, but its acceptance and incorporation into medical practice is taking longer to transpire [1].

Hypothyroidism is a disease that has been treated relatively successfully using pharmacological agents for many years. However, the treatment and management of this disease has been approached purely from a biomedical standpoint. Thyroid hormone levels that are considered inappropriate are restored using pharmacological supplementation. This management requires a life long commitment to drug therapy. In addition to this, significant proportions of patients under the current management protocol continue to experience the plethora of signs and symptoms associated with hypothyroidism, even though their thyroid hormone levels are returned to normal [26,27]. In a recent review by Roberts *et al *it was stated that approximately one fifth of hypothyroid patients are receiving an inadequate thyroxine dose and a fifth are given an excess of the synthetic thyroid hormone. Reasons for this are postulated in table 4.

**Table 4:** Potential causes of thyrotropin elevation in patients (thyroxine treated) with primary hypothyroidism

*Suboptimal dosing, inadequate prescribed dosage, dispensing error*
*Non compliance*
*Progressive decrease in endogenous thyroxine production*
*Drug and supplement interactions*
*Co-morbidities*
*Malabsorption disorders*
*Autoimmune thyroiditis*
*Previous thyroid irradiation*
*Reduced thyroxine absorption*
*Other systemic illnesses*

Another important issue that has been raised within the current literature is the suitability of modern treatment strategies for the elderly. Elderly patients often present with an extensive list of different medications that they are taking in combination. In prescribing an additional long-term pharmacological agent to the patient presenting with reduced thyroid hormone levels, practitioners must consider the issue of drug interaction. This debate is especially relevant as this group of patients makes up a substantial proportion of the hypothyroid population [33].

It is essential that thyroid hormone levels be monitored at prescribed intervals in those patients undergoing levothyroxine therapy [26]. Weetman states that overzealous supplementation can lead to an increased risk of osteoporosis in postmenopausal women and atrial fibrillation in the elderly [38]. Woeber states that in patients with pre-existing angina pectoris, treatment of hypothyroidism will result in an aggravation of symptoms in one fifth of cases. Patients with coronary heart disease run the risk of myocardial infarction some time after the initiation of levothyroxine treatment [34].

Research suggests that is it important to provide multidisciplinary care in chronic diseases (eg rheumatoid arthritis). It is further postulated that the implementation of programmes of this nature, may not only improve functioning, but may lead to improvement in disease activity [58]. The World Health Organisations (WHO) International Classification of Functioning, Disability and Health (ICF), describes a framework for understanding and structuring the impact of disease on individuals [59]. A person's functioning and disability is described as a dynamic interaction between health conditions and contextual, environmental and personal factors. Health conditions encompass disease, disorders, injuries and traumas. Contextual, environmental and personal factors describe the psychosocial elements of a person's life [60]. These guidelines align favourably with the biopsychosocial paradigm.

It seems that while current treatment protocols for hypothyroidism are quick and relatively cost efficient, there is a strong influence in form, from the biomedical model. The reductionist thinking associated with this model may be the cause of the inherent problems associated with the current treatment. As the biopsychosocial model gains credibility, it seems plausible that there may be a place for this paradigm in the management of hypothyroidism. If this disease were to be approached from a more wholistic standpoint, solutions to current management problems may be revealed. It is also feasible that if this disease were approached using a different model, factors in addition to pure biological influences may be discovered. Research into thyroid hormones and mood disorders further highlights the need for investigation in this area. A framework such as the biopsychosocial model seems to be a plausible model of inquiry.

## Conclusion

The biopsychosocial model represents the latest ideas in chronic illness management and compliments recent ideas in pain theory. It states that in order to rationalise and contend with chronic conditions, one must take into account the influence of biological, psychological and social factors. The biopsychosocial model is gaining acceptance within educational institutions and medical fields and is proving very successful in the areas in which it is applied [1]. Hypothyroidism is one chronic condition that may benefit from the application of the biopsychosocial model. Application of biopsychosocial-based interventions/therapies may help mediate some of the signs and symptoms associated with hypothyroidism. If nothing else this model represents an adjunctive framework that may facilitate a more consistent management of this chronic disease.The authors plan a randomised control trial that will seek to investigate the effect of a biopsychosocial-based treatment regime on hypothyroidism

## Authors' Contributions

BTB wrote the manuscript. All authors took part in researching for, reading and approving the final manuscript.
